# Associations of total and abdominal adiposity with risk marker patterns in children at high-risk for cardiovascular disease

**DOI:** 10.1186/s40608-015-0043-7

**Published:** 2015-03-13

**Authors:** Lawrence de Koning, Erica Denhoff, Mark D Kellogg, Sarah D de Ferranti

**Affiliations:** Calgary Laboratory Services and the Cumming School of Medicine, University of Calgary, Alberta Children’s Hospital, Room B3-724, 2888 Shaganappi Trail NW, Calgary, T3B6A8 AB Canada; Boston Children’s Hospital, 300 Longwood Ave, Boston, 02115 MA USA

**Keywords:** Waist circumference, Abdominal obesity, Biomarkers, Epidemiology, Factor analysis

## Abstract

**Background:**

While body mass index percentiles (BMI%) are commonly used to assess childhood cardiovascular risk, waist circumference percentiles (WC%) are not commonly used nor universally accepted. We tested whether BMI% or WC% should be used to identify risk factor patterns in children at high-risk for developing cardiovascular disease (CVD). A total of 107 children (8–19 years) with cardiovascular risk factors or a family history of CVD were studied. Tobacco exposure, screen-time, blood pressure and anthropometric measures were made, as well as serum risk markers. Principal component analysis (PCA) was used to identify patterns explaining risk factor variance. Multiple linear regression was used to test for associations between risk factor patterns, BMI% and WC%.

**Results:**

An adverse lipid pattern (low HDL, high triglycerides and LDL), a pro-inflammatory pattern (high ICAM and TNFαR2), a high blood pressure pattern (high SBP and DBP) and a high Lp(a) pattern were identified. Higher BMI% and WC% were associated with significantly higher levels of the lipid pattern (p < 0.05). BMI% explained 20% of variance in this pattern, whereas WC% explained 22%. When both BMI% and WC% were used together, neither BMI% nor WC% were significantly associated with the lipid pattern. However, BMI% was significantly associated with lower levels of the pro-inflammatory pattern, and WC% was associated higher levels of the pro-inflammatory pattern - together explaining 12% of variance.

**Conclusion:**

In children at high-risk for CVD, BMI% or WC% explained similar variance in an adverse lipid pattern; however, the combination of BMI% and WC% explained greater variance in a pro-inflammatory pattern than either alone. Both WC% and BMI% should both be used in anthropometric assessments of high-risk children.

## Background

Adiposity (overweight and obesity) is a powerful risk factor for cardiovascular disease (CVD) [1], and is recognized in recent obesity screening and treatment guidelines for both adults [2] and children [3,4] Adipocytes secrete fatty acids, express renin-angiotensin system components (e.g. angiotensin converting enzymes), and release inflammatory factors which advance atherosclerosis by respectively promoting hyperlipidemia and insulin resistance, high blood pressure, and vascular inflammation [5]. In particular, visceral adipocytes are extremely metabolically active, and are potent sources of these compounds - especially inflammatory factors, which are recognized as both risk factors and common pathologic features of CVD [6].

Measures of abdominal adiposity such as waist circumference (WC) indirectly assess visceral adipose tissue, and are associated with an increased risk of cardiovascular events [7]. Despite their sometimes high correlation with BMI, using them with along with BMI often improves the predictive power of models [8,9]. The importance of abdominal obesity assessment was recognized in 2008 by the College of Family Physicians of Canada, which endorsed the routine measurement of WC in adults [10]. In children, WC is assessed as a percentile for age and sex; however the role of WC percentiles in pediatric cardiometabolic risk assessment is unknown.

The objective of this study was to compare the cross-sectional associations of BMI and WC percentiles with patterns of cardiovascular risk markers – including inflammatory factors - in a population of children referred to a preventative cardiology clinic.

## Methods

This study was approved by the institutional review board of Boston Children’s Hospital, and all research conducted was consistent with declaration of Helsinki. Informed consent was obtained from parents or guardians of all participants.

A total of 150 participants aged 8–18 years of age were initially recruited from the Preventive Cardiology Clinic of Boston Children’s Hospital, Boston, Massachusetts. Medical and family history was collected by review of the clinic chart, electronic medical record and interview of a parent and patient. Family history of premature cardiovascular disease was defined as heart attack, stroke, angioplasty, stenting, coronary artery bypass grafting (CABG), peripheral arterial disease (PAD), sudden death occurring < 55 years in male and <65 years in female relatives (parents, aunts, uncles, grandparents), in a manner consistent with the National Cholesterol Education Program (NCEP) [11] and the American Academy of Pediatrics (AAP) [3,12]. All demographic information was collected by parental self-report, including exposure to tobacco smoke in the home, hours per day of television watching and computer gaming (screen time), racial background (Black, White, Other [includes Asian, Hispanic, Pacific Islander, Native American], and family history of cardiovascular disease (parent, grandparent, aunt, uncle or sibling).

### Anthropometric measures

We used the body mass index (BMI) as a measure of overall adiposity, and WC as a measure of abdominal adiposity. Height and weight were measured using a standing scale and stadiometer, and WC was measured at the level of the superior iliac crest using a tape measure. The average of two WC measures was recorded. BMI was calculated as weight in kg divided by height in meters squared. BMI percentile was determined using Centers for Disease Control (CDC) growth charts [13]. Overall obesity was defined as a BMI ≥ 95% percentile of BMI for age and sex [14,15], and abdominal obesity was defined as a WC ≥ 90^th^ percentile for age and sex [16].

Systolic (SBP) and diastolic (DBP) blood pressure were measured initially using an oscillometric cuff (Dinamap). The average of 2–3 recordings of SBP and DBP were obtained using appropriately sized cuffs using standard techniques, and converted into percentiles [17]. Elevated blood pressures (SBP > 140 mmHG, DBP > 90 mmHg) were rechecked by auscultation by experienced clinicians [17]. High blood pressure was defined as a SBP or DBP ≥ 95^th^ percentile [17].

### Laboratory measurement of risk markers

A serum sample was obtained from 107 participants after a 12-hour fast, and the following tests were performed: total cholesterol (TC), high density lipoprotein cholesterol (HDL), triglycerides (TG), low-density lipoprotein cholesterol (LDL; calculated), soluble tumor necrosis factor-alpha receptor 2 (TNF-αR2), P-selectin, intracellular adhesion molecule-1 (ICAM-1), and C-reactive protein using a high sensitivity assay (hs-CRP). Soluble tumor necrosis factor-alpha receptor 2 (TNF-αR2) is the more stable receptor of TNF-α, − which is an early inflammatory stimulator of endothelium [18]. P-selectin and ICAM-1 are both endothelial cell adhesion molecules for monocytes and lymphocytes, and C-reactive protein is a pentameric inflammatory marker that binds to bacterial cell wall components [18].

Lipids were measured enzymatically with a Hitachi 911 analyzer using reagents and calibrators from Roche Diagnostics (Indianapolis, IN, USA). The Friedewald equation was used to calculate LDL (TC – (TG/5 + HDL)) if triglycerides were less than 400 mg/dL. A direct method was used to measure LDL if triglycerides were > 400 mg/dL. Very low-density lipoprotein (VLDL) was estimated as the difference between TC and all other major lipoprotein fractions (TC – (LDL + HDL)). National Heart Lung and Blood Institute guidelines (NHLBI) were used to identify individuals with high TC (≥200 mg/dL), high LDL cholesterol (≥130 mg/dL) and low HDL cholesterol (<40 mg/dL) [19].

Lipoprotein(a) was measured with a turbidimetric assay on the Hitachi 911 analyzer (Roche Diagnostics) using reagents and calibrators from Denka Seiken (Niigata, Japan). This is the only commercial assay not affected by Kringle type 2 repeats [20]. The concentration of C-reactive protein was determined using a high-sensitivity (hs-CRP) immunoturbidimetric assay on the Hitachi 911 analyzer (Roche Diagnostics), using reagents and calibrators from Denka Seiken (Niigata, Japan). Soluble tumor necrosis factor-alpha receptor 2 (TNF-αR2), P-selectin, and intracellular adhesion molecule-1 (ICAM-1) were measured using enzyme-linked immunosorbent assays (ELISAs) from R & D Systems (Minneapolis, MN, USA).

All samples were assayed in duplicate. Samples with disparate results (coefficient of variance [CV] for duplicates > 10%) were re-assayed a third time. Day to day and within run coefficients of variance (CV) for all assays were <10%. The CVs for lipid tests were less than 2%.

### Statistical analysis

Continuous sex and race-specific (Black, White, Other) WC percentiles were interpolated from National Health and Nutrition Examination Survey (NHANES; 1999–2008) data [21] using 5-knot cubic spline regression (R; rms package). To reduce bias associated with extrapolating beyond the data, participant WC percentile above the 90^th^ percentile or below the 10^th^ percentile were rounded to 95% and 5%, respectively. The mean and standard deviation (SD) were calculated for all continuous variables. Risk markers were log-transformed prior to statistical tests and regression modeling to normalize skewed distributions. Unpaired Student’s T tests and chi squared tests were used to compare the means and proportions of each variable according to overall and abdominal obesity.

Principal component analysis (PCA) with varimax rotation was used to identify the first four independent patterns of risk markers (Proc Factor) that would explain the majority of variance in risk marker data. A continuous score was derived for each participant representing how closely their risk markers levels conformed to each pattern (Proc Score). Scores were then divided into quintiles for ease of interpretation.

Linear regression was used to assess the relationships between BMI percentile, WC percentile and quintiles of risk marker scores (Proc Reg). Partial correlation coefficients were calculated to determine the independent explanatory power of BMI and WC percentile on each score. All regression models were adjusted for exposure to cigarette smoke (Yes vs No), family history of cardiovascular disease (Yes vs No), screen time (hours), and race (Black, White vs Other). Analyses were performed in SAS ver 9.3 (Cary, NC, USA) and R version 2.15.1.

## Results

One hundred and seven participants, primarily non-Hispanic whites (82%), with an average age of 13.3 years were enrolled in the study (Table 1). Participants were at generally high risk due to high TC (58%) or hypertension (14%), as one would expect among participants recruited from a Preventive Cardiology clinic, and spent on average 2.7 hours per day watching television and playing computer games. Over half the participants had a family history of coronary heart disease (51%). All participants had hs-CRP concentrations below 1 mg/L – which denotes low cardiovascular risk in adults [22].

**Table 1:** **Participant characteristics**

	**Overall**	**Non-obese (BMI < 95%)**	**Obese (BMI ≥ 95%)**	**Non-abdominally obese (WC < 90%)**	**Abdominally obese (WC ≥ 90%)**
**% (n)**	100 (107)	57 (61)	43 (46)	67 (72)	33 (35)
**Age, y (sd)**	13.3 (2.7)	13.5 (2.9)	13.0 (2.5)	13.7 (3.0)	12.4 (2.2)*
**Race/ethnicity**					
**% White (n)**	82 (88)	90 (55)	72 (33)*	85 (61)	77 (27)
**% Black (n)**	5.6 (6)	3.3 (2)	8.7 (4)	5.6 (4)	5.7 (2)
**% Other racial group (n)**	12.1 (13)	6.6 (4)	19.6 (9)*	9.7 (7)	17.1 (6)*
**% Exposed to tobacco smoke (n)**	19 (20)	8 (5)	33 (15)*	13 (9)	31 (11)*
**% Family history of CHD (n)**	51 (55)	51 (31)	52 (24)	53 (38)	49 (17)
**Screen time, hours^(sd)**	2.7 (2.3)	2.4 (1.7)	3.1 (2.9)	2.8 (2.5)	2.5 (1.9)
**BMI percentile (n)**	80 (23)	68 (24)	97 (1)*	72 (24)	97 (2)*
**BMI, kg/m** ^**2**^ **(sd)**	25 (6)	21 (3)	30 (5)*	22 (4)	30 (6)*
**WC percentile (n)**	87 (16)	54 (23)	91 (6)*	58 (23)	95 (0)*
**WC, cm (sd)**	71 (26)	77 (11)	99 (14)*	80 (13)	100 (15)*
**Total cholesterol, mg/dL^ (sd)**	214 (50)	220 (52)	205 (47)	218 (52)	205 (47)
**% High, ≥200 mg/dL (n)**	58 (62)	59 (36)	57 (26)	58 (42)	57 (20)
**LDL cholesterol, mg/dL^ (sd)**	137 (50)	145 (50)	127 (49)*	143 (50.0)	126 (48)
**% High, ≥ 130 mg/dL (n)**	46.7 (50)	47.5 (29)	45.7 (29)	50.0 (36)	40.0 (14)
**HDL cholesterol, mg/dL^ (sd)**	53 (14)	57 (12)	48 (15)*	54 (13)	50 (17)
**% Low, < 50 mg/dL (n)**	19 (18)	7 (4)	33 (15)*	13 (9)	29 (10)*
**Triglycerides, mg/dL^ (sd)**	118 (75)	93 (57)	152 (83)*	105 (69)	147 (80)*
**% High, ≥ 130 mg/dL (n)**	34 (36)	16 (10)	57 (26)*	26 (19)	49 (17)*
**VLDL cholesterol, mg/dL^ (sd)**	24 (15)	19 (11)	30 (17)	21 (14)	29 (17)
**Lp(a)^, mmol/L (sd)**	39 (43)	39 (43)	39 (43)	40 (45)	37 (39)
**hs-CRP, mg/L^ (sd)**	0.1 (0.2)	0.1 (0.1)	0.2 (0.2)*	0.1 (0.2)	0.2 (0.2)*
**ICAM-1, ng/L^ (sd)**	295 (86)	282 (95)	312 (71)*	278 (90)	331 (65)*
**P-selectin, ng/L^ (sd)**	136 (46)	129 (46)	145 (46)	131 (46)	146 (46)
**TNF alpha, pg/L^ (sd)**	2140 (590)	2041 (501)	2271 (675)	2001 (499)	2425 (665)*
**SBP, mmHg (sd)**	112 (11)	111 (10)	114 (13)	112 (11)	113 (13)
**% SBP > 95** ^**th**^ **percentile (n)**	14 (15)	12 (7)	17 (8)	13 (9)	17 (6)
**DBP, mmHg (sd)**	63 (7)	62 (8)	64 (6)	63 (8)	63 (6)
**% DBP > 95** ^**th**^ **percentile (n)**	0 (0)	0 (0)	0 (0)	0 (0)	0 (0)

*Caption:* All continuous variables are presented as mean (standard deviation [sd]). All categorical variables and percentiles are presented as % (n). ^variables were log-transformed prior to testing for differences. *p value for comparisons < 0.05. Other racial groups were defined as Asian, Hispanic, Pacific Islander, and Native American.

Forty three percent of participants were classified as obese, whereas 33% were abdominally obese. The correlation of BMI percentile and WC percentile was 0.88. Compared to participants who were not obese, those who were obese were significantly less likely to be white and more likely to be exposed to tobacco smoke, have higher WC percentiles, triglycerides, VLDL cholesterol, hs-CRP, and ICAM-1 levels and lower LDL and HDL cholesterol. Compared to participants who were not abdominally obese, those who were abdominally obese were significantly younger, more likely to be from another racial group besides Black and White, had significantly higher BMI, triglycerides, hs-CRP, ICAM-1, TNF-αR2 but lower HDL cholesterol (Table 1).

Extraction of the first four factors using PCA identified a common clustering of risk markers suggestive of (1) an adverse lipid pattern (25% of variance explained) dominated by strong positive correlations (0.93) with triglycerides and VLDL, but a negative correlation with HDL (−0.74), (2) a pro-inflammatory pattern (14% of variance explained) having high correlations (>0.84) with ICAM-1 and TNF-αR2, (3) a high blood pressure pattern (12% of variance explained) having positive (>0.66) correlations with SBP and DBP and (4) an elevated Lp (a) pattern (10% of variance explained), having a high positive correlation with Lp(a) (0.75) (Table 2).

**Table 2:** **Varimax-rotated factor patterns**

	**Factor 1: “Adverse lipids pattern”**	**Factor 2: “Pro-inflammatory pattern”**	**Factor 3: “High BP pattern”**	**Factor 4: “High Lp(a) pattern”**
HDL	**−0.74**	0.02	−0.07	−0.06
LDL	−0.11	0.04	−0.19	0.43
Triglycerides	**0.93**	0.02	0.11	−0.18
VLDL	**0.93**	0.00	0.08	−0.17
Lp(a)	0.10	0.01	0.01	**0.75**
hs-CRP	0.37	0.33	0.47	0.11
ICAM	0.01	**0.86**	−0.19	−0.04
P-selectin	0.23	0.18	−0.18	−0.51
TNF	−0.02	**0.84**	0.21	−0.04
Systolic BP	0.06	−0.01	**0.66**	−0.50
Diastolic BP	0.08	−0.04	**0.78**	0.03
% variance explained	25%	14%	12%	10%

*Caption:* Correlations greater than 0.6 are indicated in bold.

Both BMI and WC percentiles were significantly associated with higher scores for the lipid pattern (Table 3). That is, a greater degree of overall or abdominal adiposity was associated with higher scores representing a constellation of lipid abnormalities including higher TG and VLDL, but lower HDL. Each metric explained between 20 and 22% of variance in this pattern. Neither metric was significantly associated with any of the other patterns. However when BMI and WC percentiles were included in the same model, both were significantly associated with the inflammatory pattern – but in opposite directions. In this analysis, an increase in BMI percentile was associated with a decrease in the inflammatory pattern score, whereas an increase in WC percentile was associated with an increase in the inflammatory factor score.

**Table 3:** **Association of BMI and WC percentiles with quintiles of factor scores**

	**Lipid pattern**			**Inflammatory pattern**			**BP pattern**			**Lp(a) pattern**		
	**Beta**	**P for trend**	**pR** ^**2**^	**Beta**	**P for trend**	**pR** ^**2**^	**Beta**	**P for trend**	**pR** ^**2**^	**Beta**	**P for trend**	**pR** ^**2**^
**BMI %**	0.12 (0.03)	<0.01	20%	0.01 (0.03)	0.72	5%	0.07 (0.03)	0.14	8%	0.00 (0.03)	0.98	8%
**WC %**	0.11 (0.03)	< 0.01	22%	0.05 (0.03)	0.09	8%	0.06 (0.03)	0.06	7%	0.01 (0.03)	0.84	8%
**BMI % adjusted for WC %**	0.89 (0.06)	0.14	2%	−0.14 (0.07)	0.03	5%	0.09 (0.07)	0.21	2%	−0.02 (0.07)	0.76	0%
**WC % adjusted for BMI %**	0.04 (0.05)	0.45	1%	0.16 (0.06)	0.03	8%	−0.01 (0.06)	0.84	0%	0.02 (0.06)	0.71	0%
**Total explanatory power using both metrics**			3%			12%			2%			0%
**Explanatory power gained by using both metrics**			None			4-7%			None			None

*Caption:* Beta coefficients are evaluated as change in quintile rank corresponding to a 5% increase in BMI or WC percentiles. Standard errors are presented in parentheses. All models are adjusted for exposure to cigarette smoke, family history of cardiovascular disease, hours of screen-time, white race, black race and other race. pR^2^ – partial R^2^, representing the independent explanatory power of each predictor.

## Discussion

In a pediatric population at high-risk for developing cardiovascular disease, both overall and abdominal adiposity as measured by BMI and WC percentiles were similarly associated with an adverse pattern of lipid markers including high TG, high VLDL cholesterol, and low HDL cholesterol. However when considered together, increasing WC percentile was associated with higher levels of a pro-inflammatory pattern whereas increasing BMI percentile was associated with lower levels – together explaining greater variance in this pattern than either alone.

Anthropometric assessment of cardiovascular disease risk is now routine clinical practice, mainly because adiposity is a major risk factor for conditions that lead to cardiovascular disease – including abnormal lipids, high blood pressure, and type 2 diabetes [5]. Increasing attention is now being paid to abdominal adiposity, characterized by an increased WC or waist to hip ratio, because it provides information about risk for cardiovascular disease above and beyond that provided by BMI [23]. This is particularly the case when systemic inflammation is of interest, because abdominal adiposity is more closely related to inflammation than is general adiposity [5]. Like other risk markers, inflammatory factors may be modifiable through weight loss via a prudent diet and physical activity [24]. Furthermore, the American College of Cardiology, the American Heart Association and the Canadian Cardiovascular Society recommend measuring inflammation by high-sensitivity C-reactive protein (hs-CRP) to identify patients who could obtain particular benefit from statin therapy [5,25].

As with adults, children are routinely assessed using anthropometric measures. This is done to identify individuals with abnormal growth due to malnutrition, endocrine disorders, or those at risk for premature cardiovascular disease and mortality [3]. Childhood overweight and obesity, defined respectively by the Centers for Disease Control as a BMI ≥ the 85^th^ and 95^th^ percentile for age and sex [14], is a growing problem around the world. In developed countries, the prevalence of overweight and obesity ranges from 5% in Turkey to 33% in the United States [26]. Childhood adiposity also tends to track throughout the life-course, increasing the risk that obese children will become obese adults [27]. While assessment of overall adiposity using BMI percentile is a well-accepted approach in cardiovascular risk assessment in children, measurement of abdominal adiposity has not yet become an established practice. One impediment has been the lack of age, sex and ethnic specific percentiles for measures of abdominal adiposity. Only recently have these become available through large representative cross-sectional surveys [21,28].

We compared the associations of BMI and WC percentile with risk marker patterns in a population of children referred to a pediatric cardiology clinic. In addition to more standard CVD risk factors, we also measured four inflammatory markers (TNF-αR2, P-selectin, ICAM-1, and hs-CRP) that have been associated either with excess adiposity and atherosclerosis in animal and autopsy studies, pre-clinical vascular testing and as predictors of adult cardiovascular disease events [18]. We used PCA to identify major patterns of risk markers, greatly reducing the number of individual statistical tests required, and simplifying the interpretation of results.

The first four patterns of risk markers roughly corresponded to an abnormal lipid pattern, an abnormal inflammatory marker pattern, an abnormal blood pressure pattern and an abnormal Lp(a) pattern. The lipid pattern accounted for the most variance in risk markers, and was consistent with a pattern reflecting obesity related dyslipidemia. The inflammatory pattern was characterized by increased levels of all markers except hs-CRP. We are unclear why abnormal hs-CRP levels were not associated with this pattern, however it could be due to the low level of variation in hs-CRP in this relatively young population. We found that BMI and WC percentiles were similarly associated with the lipid pattern, which is consistent with others findings in adults suggesting that both abdominal and general adiposity are associated with abnormal lipids. Neither BMI nor WC percentile was significantly associated with inflammatory, BP, or Lp(a) patterns. A different scenario emerged when both measures were used simultaneously. When BMI and WC percentiles were included in the same model, neither was significantly associated with the lipid pattern – indicating that using WC percentile in addition to BMI percentile adds no significant advantage in predicting lipid abnormalities than using either alone. However WC and BMI percentile were significantly associated with the inflammatory pattern, but in opposite directions. In this model, BMI percentile adjusted for WC percentile can be interpreted as anti-or non-inflammatory lean mass; whereas WC percentile adjusted for BMI percentile is interpreted as pro-inflammatory abdominal adipose tissue. The separation and independent consideration of these measures greatly improves the prediction of abnormal levels of inflammatory factors.

Our study supports the simultaneous use of both BMI and WC percentiles in cardiovascular risk assessment in children. Measurement of abdominal adiposity is increasingly recommended for adults, and we anticipate this to be the case for children in the future. WC is easy to obtain and as a percentile was at least as good as BMI in predicting lipid abnormalities in our study. Used in conjunction with BMI percentile, WC percentile provides clearer information on underlying cardiovascular risk due to inflammation. However, the clinical utility of using WC percentile in addition to BMI percentile needs to be further assessed in prospective studies using hard endpoints (e.g. incident type 2 diabetes, cardiovascular disease) that occur after the initial risk assessment.

Our study has several strengths. First, it reports levels of inflammatory markers in a group of children at high-risk for future early CVD. Inflammatory markers are not routinely measured in children, and there is little data in the literature on what is ‘normal’ [29]. However, it is relatively clear that inflammation is a key component of cardiovascular risk in adults, and is likely to be in children as well. Second, we used PCA to identify major risk marker patterns rather than performing individual analyses on each one. This substantially reduced the number of statistical tests we had to perform, and simplified the interpretation of the results. Third, our study is one of the first to contrast the predictive power of BMI vs WC percentiles on risk markers in children.

Our study has some limitations. First, the cross-sectional design of our study means that temporal relationships cannot be definitively established. However there is strong evidence that adipose tissue is a causal factor in the appearance of cardiovascular risk factors. Second, we did not measure dietary intake or physical activity, which means that associations between adiposity and risk marker patterns may be overestimated. Third, waist circumference percentiles were derived from aggregate percentile data that was rounded – which could lead to error in estimates. Fourth, we studied a small number of mostly non-hispanic whites, which limited both our statistical power and generalizability.

## Conclusions

In a sample of children at high-risk for developing cardiovascular disease, BMI percentile and WC percentile explained similar variance in a pattern of abnormal lipids, however WC percentile used in conjunction with BMI percentile explained the most variance in a pattern of elevated inflammatory factors. This suggests that both WC percentile and BMI percentile should be used for risk assessments of high-risk children.

## Abbreviations

WC: Waist circumference
WC%: Waist circumference percentile
BMI: Body mass index
BMI%: Body mass index percentile
CVD: Cardiovascular disease
SBP: Systolic blood pressure
DBP: Diastolic blood pressure
LDL: Low density lipoprotein cholesterol
HDL: High density lipoprotein cholesterol
VLDL: Very low density lipoprotein cholesterol
TC: Total cholesterol
TG: Triglycerides
Lp(a): Lipoprotein a
hs-CRP: High sensitivity C-reactive protein
ICAM-1: Intracellular adhesion molecule one
TNFα-R2: Tumor necrosis factor alpha receptor two
PCA: Principal component analysis
CABG: Coronary artery bypass grafting
PAD: Peripheral artery disease
NCEP: National cholesterol education program
AAP: American association of pediatrics
CDC: Centers for disease control
NHLBI: National heart lung and blood institute
ELISA: Enzyme linked immunosorbent assay
CV: Coefficient of variance
SD: Standard deviation
